# Picroside II Improves Severe Acute Pancreatitis-Induced Intestinal Barrier Injury by Inactivating Oxidative and Inflammatory TLR4-Dependent PI3K/AKT/NF-*κ*B Signaling and Improving Gut Microbiota

**DOI:** 10.1155/2020/3589497

**Published:** 2020-04-12

**Authors:** Xuehua Piao, Baohai Liu, Xiaodan Sui, Shuangdi Li, Wei Niu, Qingyu Zhang, Xuan Shi, Shusheng Cai, Ying Fan

**Affiliations:** ^1^Department of Traditional Chinese Medicine, The First affiliated Hospital, Jinzhou Medical University, Jinzhou 121001, China; ^2^Department of Gastroenterology, The First affiliated Hospital, Jinzhou Medical University, Jinzhou 121001, China; ^3^Department of Hepatology, The Affiliated Hospital of Changchun University of Traditional Chinese Medicine, 130021, China; ^4^Heart Disease Center, The Affiliated Hospital of Changchun University of Traditional Chinese Medicine, Changchun 130021, China

## Abstract

**Background:**

Picroside II exerts anti-inflammatory and antidiarrheal effects for treating the diseases associated with oxidative injury. However, its function on pancreatitis-induced intestinal barrier injury remains unclear. *Hypothesis/Purpose*. We hypothesized that picroside II will have protective effects against pancreatitis-induced intestinal barrier injury by affecting oxidative and inflammatory signaling (Toll-like receptor 4- (TLR4-) dependent phosphatidylinositol 3-kinase (PI3K), protein kinase B (AKT), and nuclear factor kappa B (NF-*κ*B)). *Study Design and Methods*. A Sprague-Dawley (SD) rat model with severe acute pancreatitis (SAP) was induced via the injection of sodium taurocholate (4% wt/vol; 1 mL/kg). All rats were divided into 3 groups: sham (CG), SAP-induced intestinal barrier injury (MG), and picroside II (PG) groups. Intestinal barrier injury was assessed by scanning electron microscopy (SEM), hematoxylin and eosin staining, and pathological scores. We measured the levels of pancreatitis biomarkers (amylase and lipase), oxidative and inflammatory signaling (TLR4-dependent PI3K/AKT/NF-*κ*B), oxidative stress marker (superoxidase dismutase (SOD), catalase (CAT), glutathione peroxidases (GPx), and malondialdehyde), and inflammatory markers (tumor necrosis factor *α* (TNF*α*), interleukin- (IL-) 1, IL-6, and IL-10) in serum and/or gut tissues. Gut microbiota composition in feces was measured by using 16S rRNA sequencing.

**Results:**

SEM showed that intestinal barrier injury was caused with the loss of intestinal villi and mitochondria destruction, and pathological scores were increased in the MG group. The levels of amylase, lipase, malondialdehyde, TNF*α*, IL-1, IL-6, TLR4, PI3K, AKT, and NF-*κ*B were increased, and the levels of SOD, GPx, CAT, and IL-10 was reduced in the MG group when compared with CG group (*P* < 0.05). Picroside II treatment inhibited the symptoms in the MG group and showed antioxidant and anti-inflammatory activities. The serum levels of picroside II had strong correlation with the levels of inflammatory and oxidative stress biomarkers (*P* < 0.05). *Picroside II treatment* increased the proportion of *Lactobacillus* and *Prevotella* and decreased the proportion of *Helicobacter* and *Escherichia_Shigella* in the model.

**Conclusions:**

Picroside II improved the SAP-induced intestinal barrier injury in the rat model by inactivating oxidant and inflammatory signaling and improving gut microbiota.

## 1. Introduction

Severe acute pancreatitis (SAP) is associated with multiple organ failure and systemic inflammatory responses with a high fatality rate of up to 15–20% [1]. Although much progression has been made in diagnostic strategies [2] and therapeutic methods [3] for SAP in recent, an effective therapeutic drug is still unavailable. Picroside II is an active constituent extracted from herbs [4, 5] and has long been used as traditional Chinese medicine for treating the diseases associated with oxidative injury and acute inflammation [5–7]. However, the role and underlying pharmacological mechanisms of picroside II in SAP are largely unknown.

Oxidative stress and the activation of inflammatory responses have been regarded to play important roles in SAP progression [8, 9]. Our previous work showed that picroside II ameliorated SAP progression by increasing antioxidant and anti-inflammatory activities of SAP-induced intestinal barrier injuries via nuclear factor kappaB- (NF-*κ*B-) dependent autophagy [10]. Actually, intestinal barrier plays an important role in the prevention of SAP risk [11], and SAP incidence can induce intestinal barrier injury [12]. The change of gut microbiota may lead to barrier failure, which is a key event contributing to the severity of gut injury [13, 14]. Toll-like receptor 4- (TLR4-) dependent phosphatidylinositol 3-kinase (PI3K)/Protein kinase B (AKT)/NF-*κ*B signaling is closely associated with oxidative stress and inflammatory responses [15, 16]. However, whether picroside II exerts its function on SAP-induced intestinal barrier injury or affects TLR4-dependent PI3K/AKT/NF-*κ*B signaling remains unknown. Therefore, in this study, we aimed to explore the related molecular mechanism for the protective effects of picroside II in the model with SAP-induced intestinal barrier injury.

## 2. Materials and Methods

### 2.1. Chemicals

Purified picroside II (>98%, CAS Number 39012-20-9, 1aS,1bS,2S,5aR,6S,6aS)-1a,1b,2,5a,6,6a-Hexahydro-6-[(4-hydroxy-3-methoxybenzoyl)oxy]-1a-(hydroxymethyl)oxireno[4, 5]cyclopenta[1,2-c]pyran-2-yl-*β*-D-glucopyranoside) was purchased from Sigma-Aldrich (St. Louis, MO, USA). HPLC analysis showed that the purity of picroside was more than 98%, and eluting time was 9.94 min ([Supplementary-material supplementary-material-1]). Sodium taurocholate (CAS Number: 145-42-6) was purchased from Sigma and dissolved in 0.9% NaCl to final concentration of 1 mg/mL.

### 2.2. Establishment of SAP-Induced Intestinal Barrier Injury

All animal-related procedures were approved by the Institutional Animal Care and Use Committee of Jinzhou Medical University (Jinzhou, China). Ninety male Sprague-Dawley (SD) rats (8 weeks old; weighing 200–220 g) were purchased from the animal center of Jinzhou Medical University (Jinzhou, China). The rats were maintained on a 12-hour-light/12-hour-dark cycle at 22°C, given a standard laboratory diet (Tecklab, Winfield, IA, USA) and water ad libitum and allowed to acclimatize for a week. SAP was established via the injection of 0.2 mL of 5% sodium taurocholate into the common biliopancreatic duct [17]. Meanwhile, in the sham group, the rats were injected with the same volume of saline solution.

### 2.3. Animal Grouping

All rats were divided into three groups, the vehicle (CG, sham rats were simultaneously injected 250 *μ*L 0.9% saline solution via tail vein, *n* = 30), model (MG, SAP rats were simultaneously injected 250 *μ*L 0.9% saline solution via tail vein, *n* = 30), and picroside II (PG, SAP rats were administrated with 25 mg/kg picroside II in 250 *μ*L 0.9% saline solution via tail vein, *n* = 30) groups.

### 2.4. Measurement of Serum Amylase and Lipase

One mL blood was withdrawn from the tail of each rat after 3-, 6-, and 24-day picroside II administration. Serum was prepared via centrifugation at 1,000 × g for 10 min and stored at −20°C for ELISA, amylase, and lipase measurement. Amylase assay kit was purchased from Abcam (ab102523, Cambridge, MA, USA), and Lipase ELISA kit was purchased from Life Science Inc. (Wuhan, China). Their activities were measured on an automatic biochemical analyzer (Dimension, Schererville, IN, USA).

### 2.5. Measurement of Biochemical Indexes in Serum

Serum levels of malondialdehyde (MDA) (MBS269473), superoxide dismutase (SOD) (#MBS080359), catalase (CAT) (#MBS775862), and glutathione peroxidase (GSPx) (#MBS049725) were also evaluated using the kits from MyBioSource, Inc. (San Diego, CA, USA). The serum levels of tumor necrosis factor *α* (TNF*α*) (ab100747), interleukin- (IL-) 1 *β* (ab100704), IL-6(ab100713), and IL-10 (ab100697) were measured by using the ELISA kits from Abcam (San Francisco, CA, USA). All biochemical indexes were measured on an automatic chemical analyzer (Hitachi, Tokyo, Japan).

### 2.6. Scanning Electron Microscopy Observation of Intestinal Barrier

For SEM processing, about 5 mm^2^ of gut mucosa were cut from each rat after 24-day picroside II administration and fixed with 1% osmium tetroxide for 2 h at 4°C. The tissues were rinsed, dehydrated in ethyl alcohol, dried with carbon dioxide, covered with gold, and examined under SEM JSM-6610lv (Jeol, Japan) with an INCA SDD X-MAX energy dispersive microanalyzer.

### 2.7. Histological Analysis of Small Intestine Tissues

Pancreatic tissues were extracted after 3-, 6-, and 24-day picroside II administration via intraperitoneal injection of phenobarbital sodium (50 mg/kg) (*n* = 10 for each group at each time). Some small intestine tissues were fixed in 4% paraformaldehyde and embedded in paraffin and remaining tissues were stored in -80°C. The embedded pancreatic tissues were cut into 2-3 *μ*m slices and stained with hematoxylin and eosin (H&E). Pancreatic edema and the numbers of inflammatory cell infiltration, bleeding, and necrotic cells were calculated. The severity of pancreatic tissue damage was assessed by using pathological score = edema score + necrosis score + inflammatory cellular infiltration score + bleeding score. Five slices were evaluated in each group.

### 2.8. Immunohistochemistry Analysis

Immunohistochemistry analysis was conducted to evaluate the expression of TLR4, PI3K, AKT, and NF-*κ*B. The paraffin-embedded tissue sections were deparaffinized and treated with hydrogen peroxide (3 *m*/*v*) for 15 min to remove endogenous peroxidase. Antigen retrieval was performed by blocking the samples in goat serum for 10 min at 22°C. The following antibodies were added and incubated 12 h at 4°C, including anti-TLR4 antibody (ab13867, 1 : 500), anti-p-PI3K p85 antibody (Abcam, ab86714, 1 : 500), anti-p-AKT antibody (ab38449, 1 : 500), and/or anti-p-NF-*κ*B antibody (ab86299) from Abcam (Cambridge, MA, USA). A biotin-labeled goat anti-rabbit IgG secondary (1 : 1000) was added followed by incubation at 37°C for 10 min. The slides were then incubated at 37°C for 10 min with peroxidase-conjugated streptavidin (Sigma, S5512). The sections were stained with 3,3′-diaminobenzidine (DAB, sigma) and counterstained with hematoxylin (Sigma). Color separation was conducted by using 2% hydrochloride and alcohol, followed by 15 min washing. Each sample was observed in five, and target protein signals were stained with brown. The positive rates were calculated as the number of positive cells/the number of total cells by using an image analyzer (Image-Pro Plus 5.1, MediaCybernetics, MD, USA).

### 2.9. Reverse Transcription-Quantitative PCR (RT-qPCR)

RNA was extracted from 5 mg small intestine using TRIzol reagent (TIANGEN, Beijing, China). cDNA was prepared by using a reverse transcription kit (Bioteck, Beijing, China) according to the manufacturer's instructions. The following primers were used: TLR4 forward primer 5′-CATGGCATTGTTCCTTTCCT-3′ and reverse primer 5′-CATGGAGCCTAATTCCCTGA-3′; PI3K forward primer 5′-TTAAACGCGAAGGCAACGA-3′ and reverse primer 5′-CAGTCTCCTCCTGCTGTCGAT-3′; AKT forward primer 5′-AAAGAGCGCATGAGTGGACG-3′ and reverse primer 5′- CGTGGTCCTCCTTGTAGTAG-3′; NF-*κ*B forward primer 5′- AGAGCAACCGAAACAGAGAGG-3′ and reverse primer 5′- TTTGCAGGCCCCACATAGTT-3′ and *β*-actin forward primer 5′-AAGTCCCTCACCCTCCCAAAAG-3′ and reverse primer 5′- AAGCAATGCTGTCACCTTCCC-3′. qPCR was conducted using SYBR-Green (Invitrogen, USA) (dilution 1 : 1000 with deionized water) for 5 min on an Applied Biosystems StepOne Plus real-time PCR machine (Applied Biosystems, Inc., CA, USA). The levels were detected and the relative mRNA levels were normalized to *β*-actin using the ΔΔCt method.

### 2.10. Western Blot Analysis

Ten mg pancreatic tissue was ground in liquid nitrogen, and total protein was extracted by using RIPA lysis (CST, Danvers, MA, USA). Protein concentration was quantified using the BCA kit (TaKaRa, Dalian, China). HRP-conjugated goat anti-rabbit IgG H&L (ab6721) secondary antibodies were from Abcam (Abcam, San Francisco, CA, USA). The proteins were separated by SDS-PAGE and transferred to the PVDF membrane in the transfer buffer at 100 V for 2–3 h. The membrane was blocked for 1 hour at ambient room temperature in 10% nonfat milk, probed with antibodies against the above primary antibodies for 2 hours at 37°C, rinsed four times with PBTB, incubated 2 hours at 37°C in secondary antibodies, and washed extensively in PBS. Images were acquired on an Odyssey CLx infrared scanner (Li-Cor-Nebraska USA). Relative protein levels were calculated by using internal reference *β*-actin.

### 2.11. Gut Microbiota Analysis

About 10 mg fresh feces were obtained from each rat after 3-, 6-, and 24-day picroside II administration. The genome of gut microbiota was isolated using a FastDNA Spin Kit (Qbiogen, Carlsbad, CA, USA). 16S rRNA was amplified by PCR using forward primer 5′-GAGAGTTTGATCCTGGCTCAG-3′ and the reverse primer 5′-GGTTACCTTGTTACGACTT-3′. Gut microbiota was analyzed by using 16S rRNA sequencing. Heat map and taxon relative abundance bar diagram was created by using custom R scripts and ggplot2.

### 2.12. Statistical Analyses

Data are presented as the mean values ± standard deviation (S.D.) and analyzed using the SPSS 21.0 software (SPSS, Inc., Chicago, IL, USA). Student's *t*-test and one-way analysis of variance (ANOVA) with post hoc Tukey's tests were used to evaluate the variables between groups. The statistical difference was significant if the value of *P* < 0.05.

## 3. Results

### 3.1. Picroside II Treatment Reduced the Activities of SAP Biomarkers

**Figure 1:**
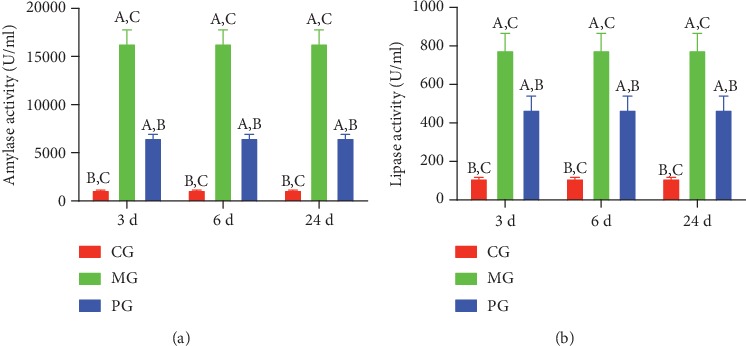
The effects of picroside II on the serum activity of amylase and lipase. (a) Serum amylase. (b) Serum lipase. ^A^*P* < 0.05 vs. the CG group, ^B^*P* < 0.05 vs. the MG group, and ^C^*P* < 0.05 vs. the PG group. All rats were divided into 3 groups, sham (CG), SAP-induced intestinal barrier injury (MG), and picroside II (PG) groups. *n* = 10 for each group.

Amylase and lipase are the potential biomarkers of pancreatitis [18]. After the establishment of SAP, the activities of serum amylase (Figure 1(a)) and lipase (Figure 1(b)) in the MG group were higher than those in the CG group (*P* < 0.05). Picroside II treatment reduced the activities of serum amylase (Figure 1(a)) and lipase (Figure 1(b)) in the PG group when compared with those in the MG group after 3-, 6-, and 24-day intervention (*P* < 0.05).

### 3.2. Picroside II Treatment Improved Intestinal Barrier Injury in the SAP-Induced Intestinal Barrier Injury

**Figure 2:**
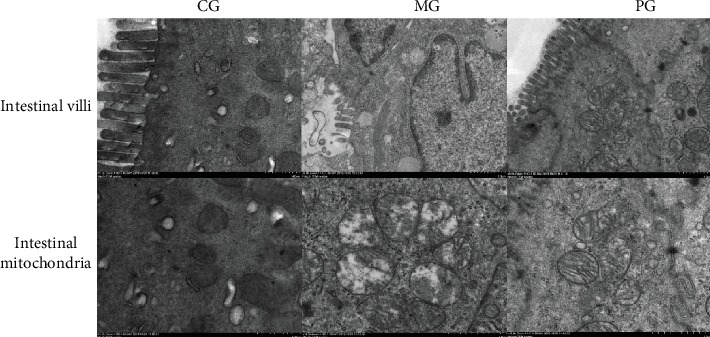
Scanning electron microscopy (SEM) observation of intestinal barrier among different groups. Intestinal villi and the structure of intestinal mitochondria were observed by using SEM after 24-day intervention.

After the establishment of SAP-induced intestinal barrier injury, the amounts of intestine villi were reduced and damaged in the MG group when compared with the CG group (Figure 2). Picroside II treatment prevented the reduction in the amounts of intestine villi when compared with the MG group (Figure 2). After the establishment of SAP-induced intestinal barrier injury, the intestinal mitochondria were expanded and structurally disordered in the MG group when compared with the CG group (Figure 2). Picroside II treatment prevented the increase in the size of intestinal mitochondria and change of mitochondria structure when compared with the MG group (Figure 2). The results suggest that picroside II treatment improves intestinal barrier injury in the SAP-induced intestinal barrier injury.

### 3.3. Picroside II Treatment Increased Antioxidant Properties in the SAP-Induced Intestinal Barrier Injury

**Figure 3:**
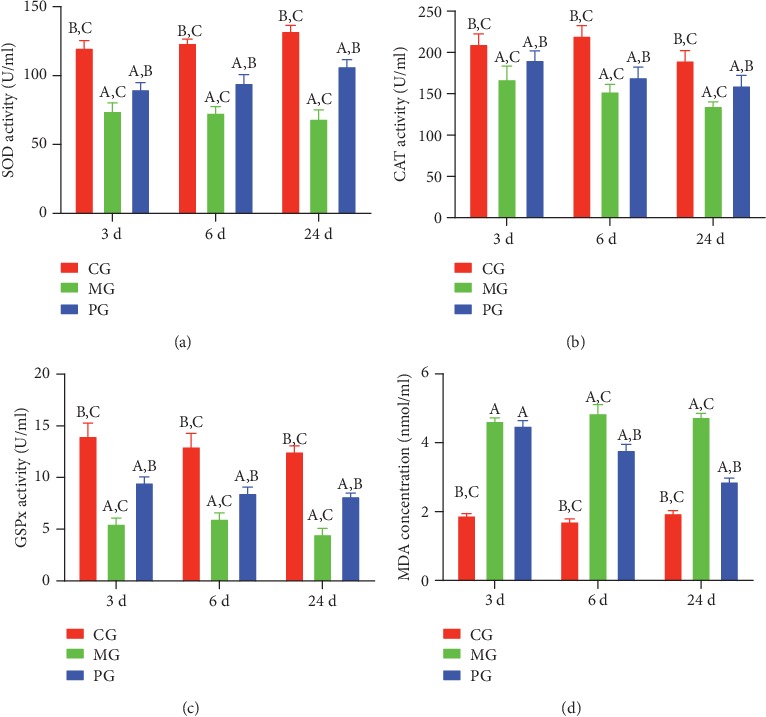
The effects of picroside II on the serum levels of oxidative stress biomarkers. (a) SOD. (b) CAT. (c) GSPx. (d) MDA. ^a^*P* < 0.05 vs. the CG group, ^b^*P* < 0.05 vs. the MG group, and ^c^*P* < 0.05 vs. the PG group. *n* = 10 for each group.

After the establishment of SAP-induced intestinal barrier injury, the serum levels of SOD (Figure 3(a)), CAT (Figure 3(b)), and GSPx (Figure 3(c)) were decreased, while the serum level of MDA (Figure 3(d)) was increased in the MG group when compared with the CG group (*P* < 0.05). Picroside II treatment increased the serum levels of SOD (Figure 3(a)), CAT (Figure 3(b)), and GSPx (Figure 3(c)) and reduced the serum level of MDA (Figure 3(d), *P* < 0.05) when compared with the MG group. The results suggest that picroside II treatment increases antioxidant properties in the SAP-induced intestinal barrier injury.

### 3.4. Picroside II Treatment Increased Anti-inflammatory Properties in the SAP-Induced Intestinal Barrier Injury

**Figure 4:**
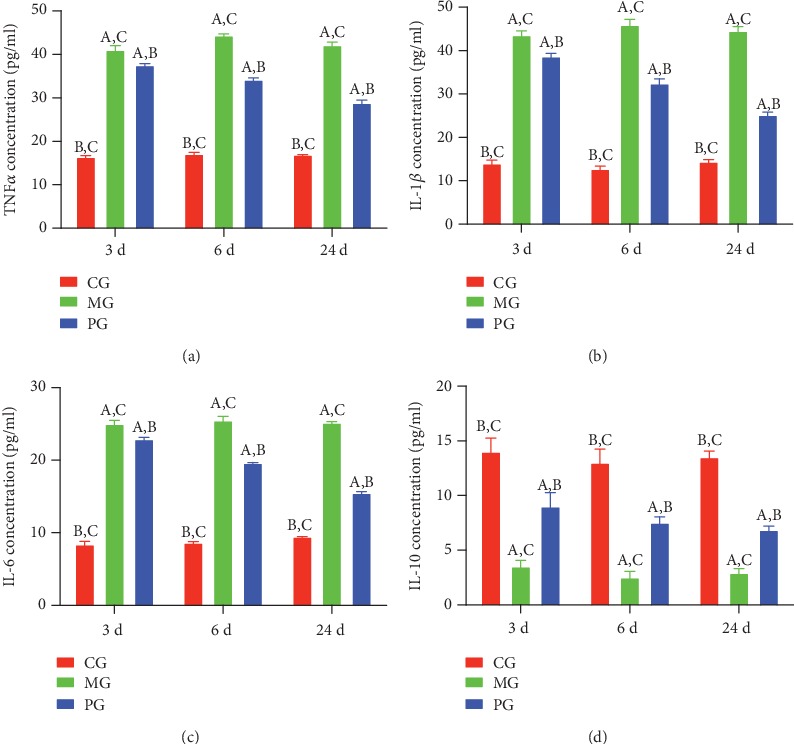
The effects of picroside II on the serum levels of inflammatory cytokines. (a) TNF*α*. (b) IL-1*β*. (c) IL-6. (d) IL-10. ^a^*P* < 0.05 vs. the CG group, ^b^*P* < 0.05 vs. the MG group, and ^c^*P* < 0.05 vs. the PG group. *n* = 10 for each group.

After the establishment of SAP-induced intestinal barrier injury, the serum levels of TNF*α* (Figure 4(a)), IL-1 (Figure 4(b)), and IL-6 (Figure 4(c)) were increased, while the serum level of IL-10 (Figure 4(d)) was reduced in the MG group when compared with the CG group (*P* < 0.05). Picroside II treatment reduced the serum levels of TNF*α* (Figure 4(a)), IL-1 (Figure 4(b)), and IL-6 (Figure 4(c)) and increased the serum level of IL-10 (Figure 4(d), *P* < 0.05). The results suggest that picroside II treatment increases anti-inflammatory properties in the SAP-induced intestinal barrier injury.

### 3.5. Picroside II Treatment Reduced Pathological Injury of SAP

**Figure 5:**
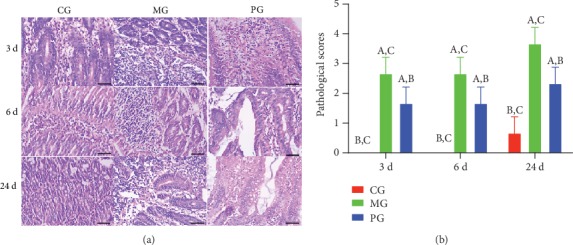
The effects of picroside II on small intestine tissues. (a) Picroside II markedly improves pathological inflammatory cell infiltration in the small intestine. (b) Pathological scores. ^a^*P* < 0.05 vs. the CG group, ^b^*P* < 0.05 vs. the MG group, and ^c^*P* < 0.05 vs. the PG group. Bar = 100 *μ*m and *n* = 10 for each group.

After the establishment of SAP-induced intestinal barrier injury, the infiltration of small intestine by immature neoplastic myeloid (Figure 5(a)) and pathological scores (Figure 5(b)) were increased in the MG group when compared with the CG group (*P* < 0.05). Picroside II treatment reduced the infiltration of neoplastic myeloid cells in small intestine and pathological scores (Figures 5(a) and 5(b), *P* < 0.05). The results suggest that picroside II treatment reduces pathological injury of SAP.

### 3.6. Picroside II Treatment Reduced TLR-Dependent PI3K/AKT/NF-*κ*B Signaling in the SAP-Induced Intestinal Barrier Injury

**Figure 6:**
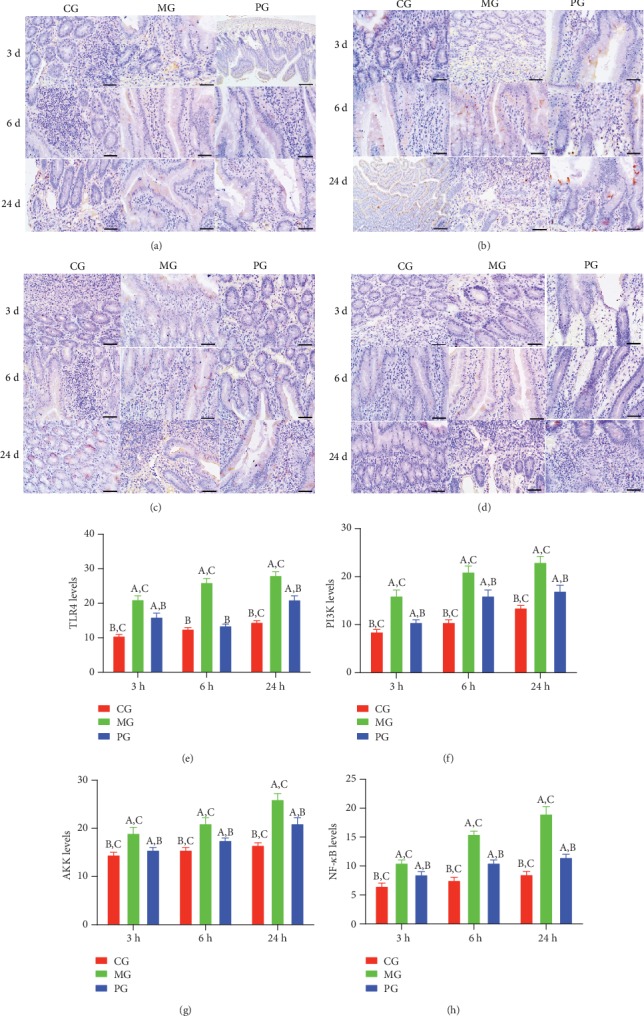
The effects of picroside II on the expression of TLR4-dependent phosphorylated PI3K/AKT/NF-*κ*B. (a) Immunohistochemical analysis of TLR4 in the colon tissues. (b) Immunohistochemical analysis of p-P3IK in the colon tissues. (c) Immunohistochemical analysis of p-AKT in the colon tissues. (d) Immunohistochemical analysis of p-NF-*κ*B in the small intestine. (e) Quantification of TLR4 levels. (f) Quantification of p-P3IK levels. (g) Quantification of p-AKT levels. (h) Quantification of p-NF-*κ*B levels in 5 different immunohistochemical images. ^a^*P* < 0.05 vs. the CG group, ^b^*P* < 0.05 vs. the MG group, and ^c^*P* < 0.05 vs. the PG group. Bar = 100 *μ*m and *n* = 10 for each group.

IHC analysis showed that the brown staining of TLR4 was increased in the MG group when compared with the CG group (Figure 6(a), *P* < 0.05). Picroside II treatment reduced the brown staining (Figure 6(a), *P* < 0.05). Similarly, the brown staining of p-P3IK was increased in the MG group when compared with the CG group (Figure 6(b), *P* < 0.05). Picroside II treatment reduced the brown staining (Figure 6(b), *P* < 0.05). The brown staining of p-AKT increased in the MG group when compared with the CG group (Figure 6(c), *P* < 0.05). Picroside II treatment reduced the brown staining (Figure 6(c), *P* < 0.05). The brown staining of p-NF-*κ*B was increased in the MG group when compared with the CG group (Figure 6(d), *P* < 0.05). Picroside II treatment reduced the brown staining (Figure 6(d), *P* < 0.05). Quantity analysis also showed that the model establishment increased the protein levels of TLR4 (Figure 6(e)), p-P3IK (Figure 6(f)), p-AKT (Figure 6(g)), and p-NF-*κ*B (Figure 6(h), *P* < 0.05). Picroside II treatment reduced the protein levels of TLR4 (Figure 6(e)), p-P3IK (Figure 6(f)), p-AKT (Figure 6(g)), and p-NF-*κ*B (Figure 6(h), *P* < 0.05) in the model. The results suggest that picroside II treatment reduces TLR-dependent PI3K/AKT/NF-*κ*B signaling in the SAP-induced intestinal barrier injury.

### 3.7. Picroside II Treatment Affected Relative mRNA Levels of TLR4 in the SAP-Induced Intestinal Barrier Injury

**Figure 7:**
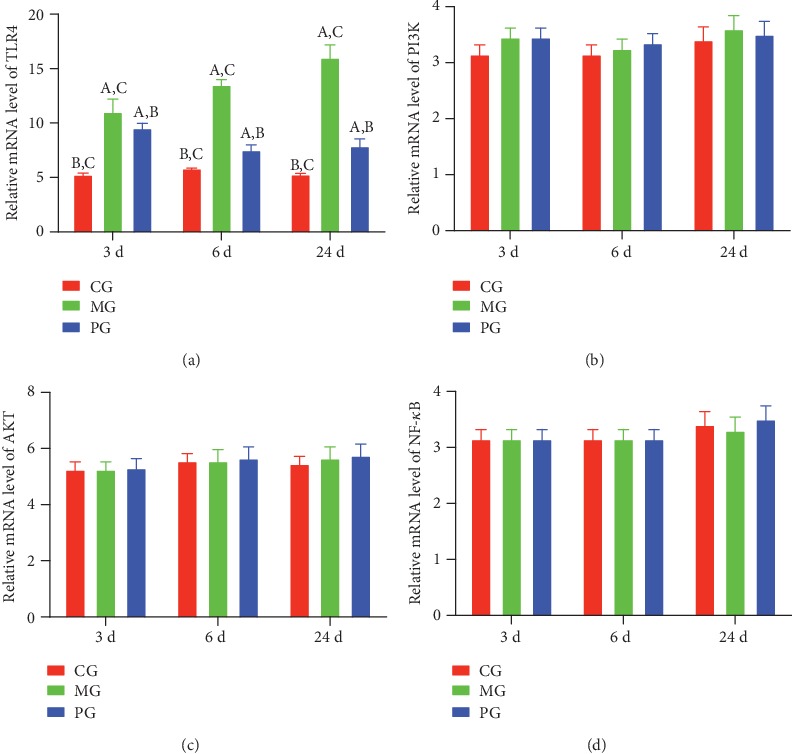
The effects of picroside II on the relative mRNA levels of TLR4-dependent PI3K/AKT/NF-*κ*B. (a) TLR4. (b) P3IK. (c) AKT. (d) NF-*κ*B. ^a^*P* < 0.05 vs. the CG group, ^b^*P* < 0.05 vs. the MG group, and ^c^*P* < 0.05 vs. the PG group. *n* = 10 for each group.

RT-qPCR analysis showed that the relative mRNA levels of TLR4 was increased in the MG group when compared with the CG group, and picroside II treatment reduced the levels (Figure 7(a), *P* < 0.05). However, relative mRNA level of P3IK changed little in the MG group when compared with the CG group and picroside II treatment did not change the level either (Figure 7(b), *P* > 0.05). Relative mRNA level of AKT also changed little in the MG group when compared with the CG group picroside II treatment did not change the level either (Figure 7(c), *P* > 0.05). There was no change for relative mRNA level of NF-*κ*B in the MG, and picroside II treatment did not cause the change either (Figure 7(d), *P* > 0.05). Picroside II treatment only affected relative mRNA levels of TLR4 but not for the level of PI3K/AKT/NF-*κ*B in the SAP-induced intestinal barrier injury.

### 3.8. Picroside II Treatment Reduced Relative Protein Levels of TLR-Dependent Phosphorylated PI3K/AKT/NF-*κ*B in the SAP-Induced Intestinal Barrier Injury

**Figure 8:**
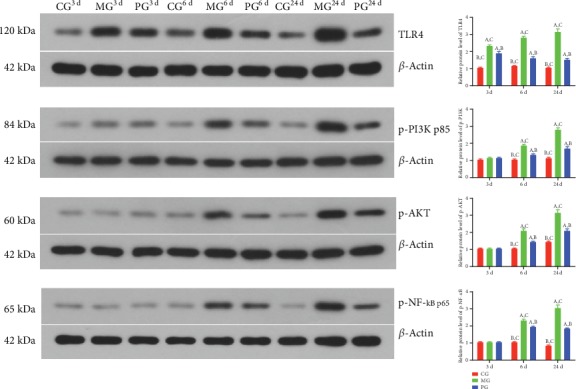
The effects of picroside II on the relative protein levels of TLR4-dependent phosphorylated PI3K/AKT/NF-*κ*B. (a) TLR4. (b) p-P3IK. (c) p-AKT. (d) p-NF-*κ*B. ^a^*P* < 0.05 vs. the CG group, ^b^*P* < 0.05 vs. the MG group, and ^c^*P* < 0.05 vs. the PG group. *n* = 10 for each group.

Western blot analysis showed that relative protein level of TLR4 was increased in the MG group when compared with the CG group, and picroside II treatment reduced the level (Figure 8(a), *P* < 0.05). Similarly, relative protein level of p-P3IK was increased in the MG group when compared with the CG group, and picroside II treatment reduced the level (Figure 8(b), *P* < 0.05). Relative protein level of p-AKT increased in the MG group when compared with the CG group, and picroside II treatment reduced the level (Figure 8(c), *P* < 0.05). Relative protein level of p-NF-*κ*B was increased in the MG group when compared with the CG group, and picroside II treatment reduced the level (Figure 8(d), *P* < 0.05). The results suggest that picroside II treatment reduces relative protein levels of TLR-dependent phosphorylated PI3K/AKT/NF-*κ*B in the SAP-induced intestinal barrier injury.

### 3.9. Picroside II Treatment Improved Gut Microbiota in the SAP-Induced Intestinal Barrier Injury

**Figure 9:**
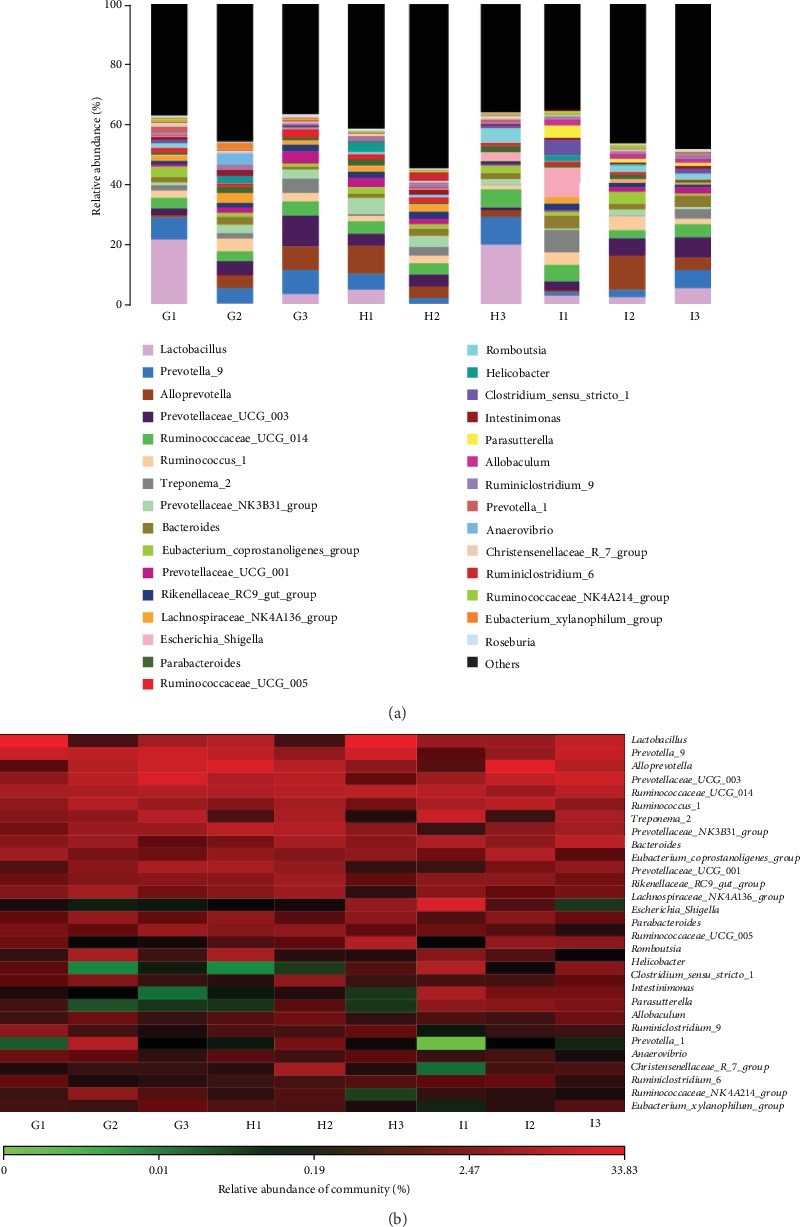
The composition of gut microbiota among different groups. (a) The proportion of gut microbiota. (b) Heat map analysis of gut microbiota changes from different treatments. G1-3 stands for the CG, MG, and PG groups at 3 d, respectively; H1-3 stands for the CG, MG, and PG groups at 6 d, respectively; I1-3 stands for the CG, MG, and PG groups at 24 d, respectively.

Bar plot showed that the proportion of Lactobacillus were decreased in the MG group, and Prevotella was decreased except of after 24-day picroside II intervention when compared with the CG group (Figure 9(a)). Picroside II treatment increased the proportion of Lactobacillus and Prevotella and decreased the proportion of *Helicobacter* and *Escherichia_Shigella* in the model (Figure 9(a)). The proportional change of major gut microbiota was provided as supplementary [Supplementary-material supplementary-material-1]. Heat map also showed that the levels of Lactobacillus were decreased, and Prevotella was decreased except of after 24-day picroside II intervention in the MG group when compared with the CG group (Figure 9(b)). Picroside II treatment increased the levels of Lactobacillus and Prevotella and decreased the proportion of Helicobacter and Escherichia_Shigella in the model (Figure 9(b)). The results suggest that picroside II treatment improved gut microbiota in the SAP-induced intestinal barrier injury.

## 4. Discussion

In the present experiment, the administration of sodium taurocholate was conducted to establish SAP-induced intestinal barrier injury, and pathological changes were found in the pancreatic tissues of MG group when compared with the CG group (Figures 5 and 6). The activities of SAP biomarker (serum amylase and lipase) were also increased (Figure 1). These results suggest that sodium taurocholate could induce SAP in rats, which had higher pathological scores in the small intestine (Figures 5 and 6). Meanwhile, oxidative stress and the inflammatory responses were also increased (Figures 3 and 4), suggesting that sodium taurocholate is an effective agent to induce SAP in the rat model and also used in other SAP studies [19, 20].

Picroside II treatment also affected the expression levels of TLR4-dependent phosphorylated PI3K/AKT/NF-*κ*B (Figure 8) but not the relative mRNA levels of PI3K/AKT/NF-*κ*B. The results suggest that picroside II may also exert its function by affecting phosphorylated situation of PI3K/AKT/NF-*κ*B via TLR4. The results were partially consistent with previous report that hydrogen sulfide mitigates SAP via PI3K/AKT/NF-*κ*B pathway [21]. The result also suggests that picroside II may exert its antioxidant and anti-inflammatory properties in SAP-induced intestinal barrier injury by inactivating MAPK/NF-kappaB signaling. Picroside II may be an effective compound to prevent SAP development with fewer side effects without toxicity as natural products [22].

The improvement of antioxidant and anti-inflammatory properties is the potential approaches for preventing SAP progression [23, 24]. In the present work, picroside II protected rats against SAP development by increasing antioxidant and anti-inflammatory capacities (Figures 3 and 4). Picroside II may be affective to increase the antioxidant and anti-inflammatory activities in the prevention of SAP progression. SAP-induced intestinal barrier injury may cause the change of gut microbiota (Figure 9) and result in intestinal barrier infection and damage (Figure 1). Picroside II treatment improved gut microbiota (Figure 9) and prevented the intestinal barrier injury and damage of intestinal mitochondria (Figure 1). The results may be also associated with the improvement of antioxidant and anti-inflammatory in the SAP-induced intestinal barrier injury model. Picroside II treatment increased the proportion of Lactobacillus and Prevotella and decreased the proportion of *Helicobacter* and *Escherichia_Shigella*. Lactobacillus species exerts protective effects on intestinal integrity and immune responses of the animal infected with Clostridium [25]. The improvement of anti-inflammatory status has been reported to be followed by the increase in the abundance of Prevotella [26]. Intestinal barrier injury is closely associated with numerous factors, such as bacterial infection, inflammation, and mechanical damage; all of which can be caused by *Helicobacter* and *Escherichia_Shigella* infection [27, 28]. Thus, picroside II treatment may ameliorate intestinal barrier injury by improving the proportion of gut microbiota.

## Data Availability

All data related to this paper may also be requested from the corresponding authors (with a lead contact at the Email: liubaoh627@163.com).

## Abbreviations

AKT: Protein kinase B
CAT: Catalase
GPx: Glutathione peroxidases
IL-1: Interleukin-1
MDA: Malondialdehyde.
NF-*κ*B: Nuclear factor kappa B
RT-qPCR: Reverse transcription-quantitative PCR
PI3K: Phosphatidylinositol 3-kinase
Picroside II: 1aS,1bS,2S,5aR,6S,6aS)-1a,1b,2,5a,6,6a-Hexahydro-6-[(4-hydroxy-3-methoxybenzoyl)oxy]-1a-(hydroxymethyl)oxireno[4,5]cyclopenta[1,2-c]pyran-2-yl-*β*-D-glucopyranoside)
SAP: Severe acute pancreatitis
SD: Sprague-Dawley
SEM: Scanning electron microscopy
SOD: Superoxidase dismutase
TLR4: Toll-like receptor 4
TNF*α*: Tumor necrosis factor *α*.

## Supplementary Materials

Supplementary Materials Table S1: the proportional change of major gut microbiota (100%). Figure S1: HPLC analysis of the purity of picroside II standard. Waters e2695-2998 chromatography system was purchased from Waters Corporation. The following HPLC conditions were set: chromatographic column, Agilent Zorbax Extend C18 (4.6 mm × 150 mm, 5 *μ*m); mobile phase, acetonitrile water-phosphoric acid (13 : 87 : 0.1); flow rate, 1.0 mL/min; column temperature, 35°C; injection volume, 10 *μ*L; and detection wavelength was 275 nm. Picroside II standard was dissolved in methanol to make a mixed solution containing 80 *μ*g per 1 mL. Ten *μ*L of picroside II standard solution was injected into the liquid chromatograph.
