# Supplementary material for: Picroside II Improves Severe Acute Pancreatitis-Induced Intestinal Barrier Injury by Inactivating Oxidative and Inflammatory TLR4-Dependent PI3K/AKT/NF-κB Signaling and Improving Gut Microbiota
Source: Oxid Med Cell Longev. 2020 Apr 12;2020:3589497. doi: 10.1155/2020/3589497 (PMC7174951; doi:10.1155/2020/3589497)
Supplement: Supplementary Materials — Table S1: the proportional change of major gut microbiota (100%). Figure S1: HPLC analysis of the purity of picroside II standard. Waters e2695-2998 chromatography system was purchased from Waters Corporation. The following HPLC conditions were set: chromatographic column, Agilent Zorbax Extend C18 (4.6 mm × 150 mm, 5 μm); mobile phase, acetonitrile water-phosphoric acid (13 : 87 : 0.1); flow rate, 1.0 mL/min; column temperature, 35°C; injection volume, 10 μL; and detection wavelength was 275 nm. Picroside II standard was dissolved in methanol to make a mixed solution containing 80 μg per 1 mL. Ten μL of picroside II standard solution was injected into the liquid chromatograph. [file 3589497.f1.docx]

**Table S1**. The proportional change of major gut microbiota (100%).

| Taxonomy | G1 | G2 | G3 | H1 | H2 | H3 | I1 | I2 | I3 |
| --- | --- | --- | --- | --- | --- | --- | --- | --- | --- |
| Lactobacillus | 34.00 | 0.85 | 5.42 | 7.91 | 0.87 | 32.66 | 4.78 | 4.11 | 8.64 |
| Prevotella_9 | 11.89 | 7.80 | 12.81 | 8.45 | 2.12 | 10.53 | 1.41 | 3.66 | 9.45 |
| Alloprevotella | 1.41 | 6.73 | 12.59 | 14.87 | 3.60 | 6.15 | 1.25 | 18.14 | 6.47 |
| Prevotellaceae_UCG_003 | 3.43 | 7.38 | 16.34 | 6.15 | 1.60 | 6.38 | 5.01 | 9.18 | 10.95 |
| Ruminococcaceae_UCG_014 | 5.66 | 5.46 | 7.18 | 6.65 | 9.51 | 6.07 | 8.49 | 4.00 | 6.82 |
| Ruminococcus_1 | 3.63 | 6.66 | 4.58 | 2.80 | 2.26 | 3.98 | 6.50 | 7.43 | 2.83 |
| Treponema_2 | 2.98 | 3.00 | 8.52 | 0.96 | 0.40 | 4.64 | 11.97 | 0.65 | 5.39 |
| Prevotellaceae_NK3B31_group | 2.12 | 4.09 | 4.57 | 8.36 | 3.56 | 5.53 | 0.78 | 3.00 | 4.59 |
| Bacteroides | 3.11 | 4.18 | 1.54 | 2.15 | 3.33 | 4.21 | 6.83 | 2.94 | 2.27 |
| [Eubacterium]_coprostanoligenes_group | 5.29 | 2.13 | 1.86 | 3.66 | 3.81 | 2.04 | 2.07 | 6.39 | 1.07 |
| Prevotellaceae_UCG_001 | 1.12 | 2.92 | 6.35 | 5.70 | 0.67 | 2.97 | 0.77 | 2.12 | 3.06 |
| Rikenellaceae_RC9_gut_group | 1.87 | 2.42 | 3.36 | 3.05 | 1.61 | 3.85 | 3.51 | 3.03 | 1.69 |
| Lachnospiraceae_NK4A136_group | 2.98 | 5.13 | 2.13 | 2.98 | 0.57 | 3.69 | 3.42 | 1.37 | 1.87 |
| Escherichia_Shigella | 0.32 | 0.11 | 0.14 | 0.22 | 4.30 | 0.19 | 16.30 | 0.95 | 0.06 |
| Parabacteroides | 1.53 | 3.50 | 1.61 | 3.46 | 3.61 | 0.94 | 1.10 | 2.68 | 1.39 |
| Ruminococcaceae_UCG_005 | 2.46 | 1.36 | 4.60 | 2.92 | 1.53 | 2.66 | 1.96 | 1.04 | 0.37 |
| Romboutsia | 1.84 | 0.21 | 0.29 | 1.00 | 7.46 | 1.02 | 0.19 | 3.27 | 2.92 |
| Helicobacter | 0.60 | 5.21 | 0.81 | 5.60 | 0.41 | 0.37 | 3.21 | 0.99 | 0.19 |
| Clostridium_sensu_stricto_1 | 1.47 | 0.01 | 0.12 | 0.01 | 1.22 | 0.04 | 8.14 | 0.21 | 2.53 |
| Intestinimonas | 1.47 | 2.48 | 0.67 | 0.45 | 0.76 | 2.65 | 0.96 | 0.77 | 1.26 |
| Parasutterella | 0.31 | 0.18 | 0.02 | 0.13 | 0.07 | 0.29 | 6.64 | 1.89 | 1.78 |
| Allobaculum | 0.91 | 0.03 | 0.07 | 0.07 | 0.07 | 0.97 | 3.62 | 2.62 | 2.16 |
| Ruminiclostridium_9 | 0.77 | 1.66 | 0.69 | 1.09 | 0.62 | 1.38 | 1.10 | 0.67 | 1.53 |
| Prevotella_1 | 3.61 | 0.75 | 0.33 | 0.99 | 1.75 | 0.68 | 0.14 | 0.58 | 0.65 |
| Anaerovibrio | 0.03 | 6.50 | 0.20 | 0.13 | 0.26 | 1.71 | 0.00 | 0.18 | 0.09 |
| Christensenellaceae_R_7_group | 2.00 | 1.33 | 0.57 | 1.25 | 1.46 | 0.59 | 0.73 | 0.78 | 0.26 |
| Ruminiclostridium_6 | 0.39 | 0.58 | 0.70 | 0.47 | 0.41 | 4.29 | 0.02 | 0.79 | 0.87 |
| Ruminococcaceae_NK4A214_group | 1.63 | 0.28 | 0.48 | 0.69 | 1.20 | 0.67 | 1.51 | 1.44 | 0.45 |
| [Eubacterium]_xylanophilum_group | 0.73 | 3.18 | 1.25 | 0.51 | 0.06 | 0.82 | 0.65 | 0.41 | 0.24 |
| Roseburia | 0.92 | 0.69 | 1.68 | 1.02 | 0.33 | 0.78 | 0.11 | 0.45 | 1.04 |

Note: G1-3, stands for the CG, MG and PG groups at 3 d, respectively; H1-3, stands for the CG, MG and PG groups at 6 d, respectively; I1-3, stands for the CG, MG and PG groups at 24 d, respectively.

**Figure S1**. HPLC analysis of the purity of picroside II standard. Waters e2695-2998 chromatography system was purchased from Waters Corporation. The following HPLC conditions were set: Chromatographic column, Agilent Zorbax Extend C18 (4.6 mm × 150 mm, 5 μm); mobile phase, acetonitrile water-phosphoric acid (13: 87: 0.1); flow rate, 1.0 mL / min; column temperature, 35 °C; injection volume, 10 μL and detection wavelength was 275 nm. PicrosideⅡ standard was dissolved it in methanol to make a mixed solution containing 80 μg per 1 mL. Ten μL of picroside II standard solution was injected into the liquid chromatograph.
